# The Effect of Cataract on Color Vision Measurement with the Low-Vision Cambridge Colour Test: Providing an Adjustment Factor for Clinical Trials

**DOI:** 10.1016/j.xops.2022.100153

**Published:** 2022-04-08

**Authors:** Jasleen K. Jolly, Luke Pratt, Aman K. More, Jennifer Kwan, Rebecca L. Jones, Robert E. MacLaren, Sher Aslam

**Affiliations:** 1 Nuffield Laboratory of Ophthalmology, Nuffield Department of Clinical Neurosciences, University of Oxford, Oxford, United Kingdom; 2 Oxford Eye Hospital, Oxford University Hospitals NHS Foundation Trust, Oxford, United Kingdom; 3 Vision and Eye Research Institute, Anglia Ruskin University, Cambridge, United Kingdom

**Keywords:** Cambridge colour text, Cataract, Color vision, Cones, Outcome measure, CCT, Cambridge Colour Test, CI, confidence interval, lvCCT, low-vision Cambridge Color Test

## Abstract

**Purpose:**

To quantify the effect of cataract on color vision as measured by the low-vision Cambridge Colour Test (lvCCT; Cambridge Research Systems) and to understand whether different types and severities of cataract have different effects on color vision.

**Design:**

Cohort study.

**Participants:**

Patients aged 18 to 95 undergoing routine cataract surgery at the Oxford Eye Hospital.

**Methods:**

The lvCCT was performed to measure color sensitivity in both eyes both before and after surgery. The crystalline lens was examined and graded according to the Lens Opacities Classification System III to determine the type and severity of cataract. Measures of repeatability were performed for the data to explore test–retest bias using Bland–Altman analysis. The Wilcoxon signed-rank test was performed to assess the effect of cataract on color vision by comparing control and surgical test measurements. Three multiple linear regressions were performed to relate cataract grading or severity to color vision measurements.

**Main Outcome Measures:**

Color discrimination along each of the protan, deutan, and tritan confusion lines.

**Results:**

The Wilcoxon signed-rank test showed a statistically significant difference in both the protan (*P* = 0.024) and tritan (*P* = 0.020) axes on comparison of control and surgical test measurements. As severity of cataract increased, color vision sensitivity was affected more greatly, and nuclear sclerotic cataract showed the most profound effect on color vision sensitivity in the lvCCT; however, the linear regression models showed that these observations did not reach statistical significance.

**Conclusions:**

Cataract surgery has a statistically significant effect on color vision in both the protan and tritan axes. The effects of specific subtypes of cataract and different severities could not be elucidated because of the high prevalence of patients with mixed cataract. The lvCCT color sensitivity measurements are used regularly as outcome measures in clinical gene therapy trials involving vitreoretinal surgery, and vitrectomy accelerates cataract formation. Therefore, it is important to quantify the effect of cataract on color vision measurements so that it may be taken into account when used as an outcome measure in clinical trials. We were unable to derive a precise correction factor for cataract on color vision measurements.

Color vision is interpreted as a measure of cone function as well as ganglion cell processing. It has been used for diagnostic and disease classification purposes for various ocular pathologic features.^1^ Perception of color vision depends on anatomic components such as ocular media clarity and cone photoreceptor viability, as well as higher neural processing pathways. Color vision is a recognized outcome measure in clinical trials for novel ocular therapies, necessitating the need for precise measurement.^2^ Cataract is the most common ocular condition and is a normal part of the aging process. In addition, cataract forms in response to vitreoretinal procedures, including gene therapy.^3^^,^^4^ To monitor the outcome of these procedures accurately, the impact of cataract on color vision measurement must be accounted for.

Cataracts form when degeneration or disruption occurs either in the anatomic structure of the lens or to the biochemical processes within the lens. With age, an increase in optical density of the lens occurs. Dysregulation of lens metabolism leads to changes to lens crystallins with deposition of yellow pigment. Water accumulation within the lens further disrupts the ordered arrangement of crystallins that, together with increased vacuolation of lens fibers and formation of crystallin aggregates, causes a reduction in light transmission.^5^ The pathophysiologic features behind cataractous lenses are thought to be associated with the aggregation of crystallins producing high–molecular-weight complexes that account for the changes in both light scattering and opacity.^6^ In particular, short-wavelength light transmission is reduced disproportionately with increasing age as a result of increased lens senescence.^7^^,^^8^

Three main types of cataract have been identified according to anatomic location. These can occur alone or in combination. Nuclear sclerotic cataract forms as part of the aging process and involves the central part of the lens. This type of cataract progresses slowly, but its formation also can be accelerated, depending on lifetime accumulation of free-radical damage and crystallin misfolding.^9^ It typically is associated with a yellowing of the lens and may cause a myopic shift in refraction as a result of refractive index changes in the lens.^10^

A cortical cataract occurs when the outer lens cortex becomes opaque and often is viewed with an ophthalmoscope as white spokes of a wheel. Spoking cataracts often occur when changes in the fluid contained in the periphery of the lens occur that cause fissuring and formation of water clefts and vacuoles in the lens cortex. Risk of formation of cortical cataracts is higher with sun exposure and ultraviolet B radiation in particular.^11^ Such cataracts lead to more problems with glare and scattering of light, particularly at night.

Posterior subcapsular cataracts occur when opacification of the posterior lens capsule and posterior lens cortex occurs that may affect vision disproportionately compared with its appearance.^9^ The prevalence of posterior subcapsular cataracts is higher in certain metabolic conditions, such as diabetes, or after use of corticosteroids and is thought to be the result of the accumulation of osmotically active polyols, such as sorbitol in the lens fibers and disruption to oxidative mechanisms.^5^^,^^11^ The faster progression of posterior subcapsular cataracts and their greater impact on light scattering mean that these cataracts subjectively have the greatest effect on vision.

Anecdotal and subjective reports on color vision outcomes after cataract surgery mention that postoperative colors are more vibrant and that whites are whiter rather than being tinged in a dull beige overtone. To quantify color perception, a reliable test capable of being used with a range of visual acuities, able to detect acquired color vision loss, and sensitive enough to detect small changes in hue and saturation is required. This led to the development of computerized testing of color vision.

Color discrimination in this study was conducted with the use of the low-vision version Trivector test of the Cambridge Colour Test (CCT; Metropsis, Cambridge Research Systems). The CCT is a computerized test of color discrimination that is used for the assessment of color vision in both research and clinical practice.^12^ It is based on the traditional pseudoisochromatic test plates but incorporates the principles of Chibret and Stilling by varying the chromaticity of the target stimulus with varying luminance of neutral-hued elements.^13^ The aim of the test is for the subject to be able to identify the lowest saturation when a chromatic difference still can be detected against the neutral-hued distractors. The added advantage of it being a computerized test allows for greater discernibility of an individual test subject's color discrimination by using a staircase method of measuring thresholds and provides precise variation of chromatic differences that can be tailored to the subject’s performance.^14^ Furthermore, by randomly generating combinations of stimulus and background elements, the computerized CCT prevents systematic bias through a learning effect demonstrating good repeatability.^12^

The Trivector subtest provides an estimate of discrimination thresholds along the protan, deutan, and tritan confusion lines. Each of these 3 parameters has been optimized to correspond to the sensitivity of L, M, and S cones, respectively. Since its release by Cambridge Research Systems Ltd in 2000, the CCT has demonstrated its ability as a diagnostic and monitoring tool sensitive enough for normal trichomats as well as discriminatory enough for monitoring patients’ gradual losses in color vision as a result of congenital or acquired color vision deficiencies.^7^^,^^12^^,^^15^ The low-vision version is currently being used as an outcome measure in an ongoing phase II gene therapy trial because it can be performed regardless of the level of visual acuity.^16^

Our prospective observational cohort study aims to measure the reduction in color vision sensitivity quantitatively using the CCT because of the presence of cataract. A cohort of healthy patients across a wide age range undergoing cataract surgery was recruited for color sensitivity testing using the CCT. The color vision tests were performed before and after cataract surgery. Our secondary objective was to understand how different types and grades of cataract affect color vision measurements by relating the preoperative grading of the cataracts to color vision measurements. The grading of cataracts was completed by visual assessment of the crystalline lens using a slit lamp that then was compared with the Lens Opacities Classification System III grading system to establish both the type and grade of cataract present.^17^

## Methods

### Participants

Participants were recruited from patients 18 to 95 years of age attending for routine cataract surgery at the Oxford Eye Hospital. Ethical approval was obtained from the Health Research Authority and Research Ethics Committee (reference no.: 17/LO/1412), and the study complied with the tenets of the Declaration of Helsinki. All participants provided informed consent. The baseline visit was scheduled at the time of the preoperative assessment appointment, and follow-up at the time of the routine postoperative check was conducted 4 to 6 weeks after cataract surgery. Participants with concurrent comorbidity or medication use that could affect color vision or with known congenital color vision defect were excluded. Patients who experienced surgical complications during surgery also were excluded to reduce confounding factors for analysis of the color vision scores. Participant demographics are shown in Table 1. Of the 50 participants, 11 were removed after the first assessment because of surgical complications or the failure to return for the postoperative visit.

**Table 1:** Clinical Characteristics of Study Patients

Patient Characteristics	All Eyes	Surgical Eyes	Control Eyes
Age (yrs)			
Before surgery (n = 50)	74 (51–91)		
After surgery (n = 39)	73 (51–91)		
Male sex (%)			
Before surgery	36		
After surgery	31		
Time between surgery and postoperative appointment (days)	33 (24–65)		
Cataract morphologic features (no.)			
Nuclear		43	13
Cortical		19	4
Posterior subcapsular		13	0
Pseudophakic		N/A	25

N/A = not applicable.

Data are presented as mean (range) unless otherwise indicated.

All participants underwent an initial ophthalmic examination that included visual acuity, and Lens Opacities Classification System III grading of the cataract was conducted by a single observer (S.A.).^17^ The eye undergoing cataract surgery was assigned as the treated eye, and the contralateral eye was assigned as the control eye. All operated eyes underwent implantation of a ultraviolet light-absorbing, hydrophobic acrylic monofocal intraocular lens (TECNIS iTec PCB00; Abbott Medical Optics, Inc) aiming for emmetropia. Visual assessments were repeated at the follow-up visit.

### Color Vision Testing

The low-vision version of the CCT was used in which 4 homogenous discs appear on the Cambridge Research Systems liquid crystal display (LCD) Display++ monitor and 1 of the discs differs in chromaticity from the others. The 3 other discs have a neutral hue without any color. The patient’s task is to identify the disc that differs from the others in color. If the patient identified the correct disc, the stimulus saturation decreased at next presentation. If the patient gave an incorrect answer, the saturation increased. The low-vision CCT procedure elucidates the minimum saturation required to discriminate the target against luminance noise. Higher scores indicated a decreased color sensitivity because the minimum chromaticity needed for the disc to be discriminated was higher. The procedure continued until a saturation threshold was determined for each vector.

The stimulus pattern consisted of 4 discs arranged in a diamond configuration on a 2 cd/m^2^ neutral background 1.5 m away from the observer. Only 1 of the discs differed in chromaticity (CIE [1976] u′v′ chromaticity diagram) from the other 3 that remained neutral in hue (*u*′ = 0.211, *v*′ = 0.474) but with varying luminance (between 6 and 26 cd/m^2^). Each stimulus was presented for a maximum duration of 60 seconds, and participants were given standardized verbal instruction to determine which disc differed in chromaticity from the other 3 distractors and to respond accordingly with the arrow keys on a keyboard held by the participant (4-alternative forced choice). A response, whether correct or incorrect, ended the stimulus. A nonresponse was taken to be an incorrect response. A staircase method with 6 of 7 reversals was used to determine the discrimination threshold along each of the protan, deutan, and tritan confusion lines in u′v′ units. Each staircase began with high saturation, and the chromaticity of the target disc relative to the other 3 of neutral hue varied so that chromatic contrast is halved after a correct response and doubled after an incorrect response. Tests were conducted 3 times for each eye (surgical or control) at each visit (before and after surgery).

### Statistical Analysis

All statistical analyses were carried out with SPSS software version 27 (IBM Software). Primary analysis of the raw color vision measurement data was carried out to determine if the data followed a normal distribution. Because it did not, the values were logged, and Bland–Altman analysis was undertaken to determine the limits of repeatability for multiple testing.^18^ The coefficient of repeatability was calculated using a 1-way repeated-measures analysis of variance.^19^ Data were removed from the cohort for 2 patients because it was noted that they had not understood the aim of the task and therefore had achieved the highest score by default at both visits in both eyes.

The effect of cataract on color vision was assessed using the Wilcoxon signed-rank test, comparing the difference in color score between visits 1 and 2 in the treated eyes versus the control eyes. To quantify the effect of cataract grade and severity, 3 multiple linear regressions for the change in protan, deutan, and tritan were calculated, respectively. The level of statistical significance was set at *P* ≤ 0.05, using correction for multiple testing when appropriate.

## Results

### Learning Effect and Repeatability Measures of the Trivector Subtest

**Figure 1:**
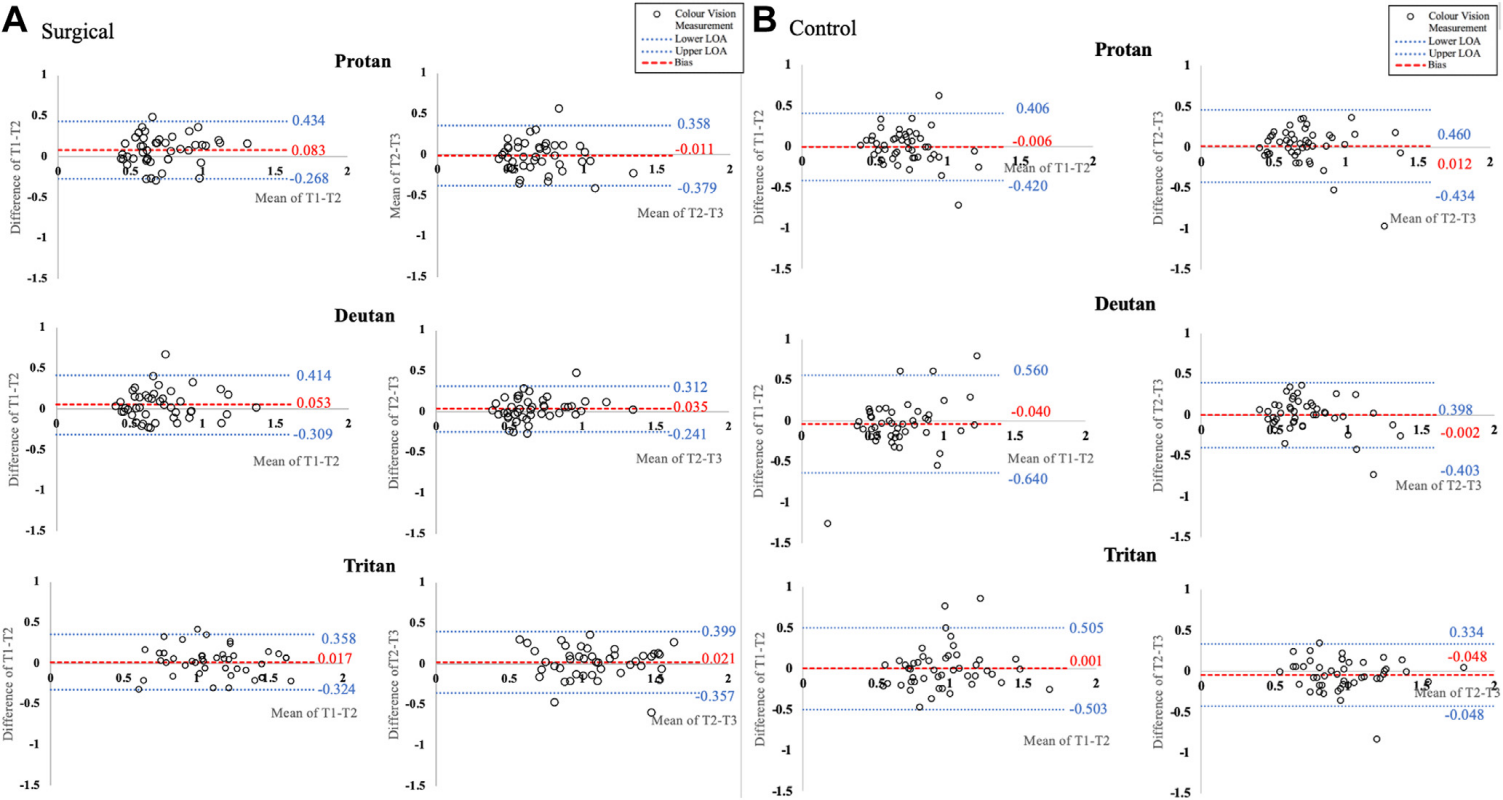
Bland–Altman plots showing color vision testing results at the preoperative (baseline) visit. The (**A**) surgical eye was the eye with cataract that was undergoing cataract surgery and (**B**) the control eye was the other eye. Bland–Altman graphs are shown separately for protan, deutan, and tritan axes. Mean differences are indicated with red dashed lines, and limits of agreement (LOA) are indicated with blue dotted lines. Differences between test 1 (T1) and test 2 (T2) indicate a learning effect takes place because of the wider LOA, so T2 and test 3 (T3) are used to investigate impact of cataract, with the first test allowing for learning to take place. Color vision measures are in × 1000 CIE1976 luv units.

Bland–Altman analysis was conducted to analyze the repeatability of the data. Plots were produced to compare the repeatability between test 1 to test 2 and test 2 to test 3 for each of the Trivector measurements (Fig 1A). Data were grouped into surgical and control eye groups, and then preoperative and postoperative were the 2 time points.

Multiple 1-way repeated-measures analyses of variance were conducted using both eye and time points as factors for investigation in the Bland–Altman analysis to calculate coefficients of repeatability (Table 2). The coefficient of repeatability is a measure of test repeatability, with a smaller number representing greater repeatability. To analyze the presence of a learning effect within the data, the test 1 versus test 2 and test 2 versus test 3 coefficients of repeatability were compared within both the surgical and control eyes separately. A slight learning effect was seen between test 1 and test 2 within the control eye; therefore, test 1 was discarded from further statistical analyses performed. On this basis, we considered the difference between test 1 and test 2 to be a learning effect and the difference between test 2 and test 3 to be a measure of test–retest repeatability.

**Table 2:** Coefficients of Repeatability

	Control Eye				Surgical Eye			
	Test 1 vs. Test 2		Test 2 vs. Test 3		Test 1 vs. Test 2		Test 2 vs. Test 3	
	Log	Antilog	Log	Antilog	Log	Antilog	Log	Antilog
Before surgery								
Protan	0.41	2.58	0.45	2.80	0.35	2.24	0.37	2.35
Deutan	0.60	3.99	0.40	2.52	0.26	2.30	0.28	1.89
Tritan	0.50	3.19	0.38	2.41	0.34	2.18	0.38	2.41
After surgery								
Protan	0.38	2.41	0.48	3.02	0.48	3.02	0.36	2.29
Deutan	0.33	2.13	0.61	4.10	0.52	3.30	0.49	3.07
Tritan	0.28	1.89	0.36	2.30	0.48	3.02	0.38	2.41

One-way repeated measures analyses of variance calculated for each test–retest group of the Bland–Altman analysis were used to produce coefficients of repeatability.

### Effect of Cataract Removal on Color Sensitivity

**Figure 2:**
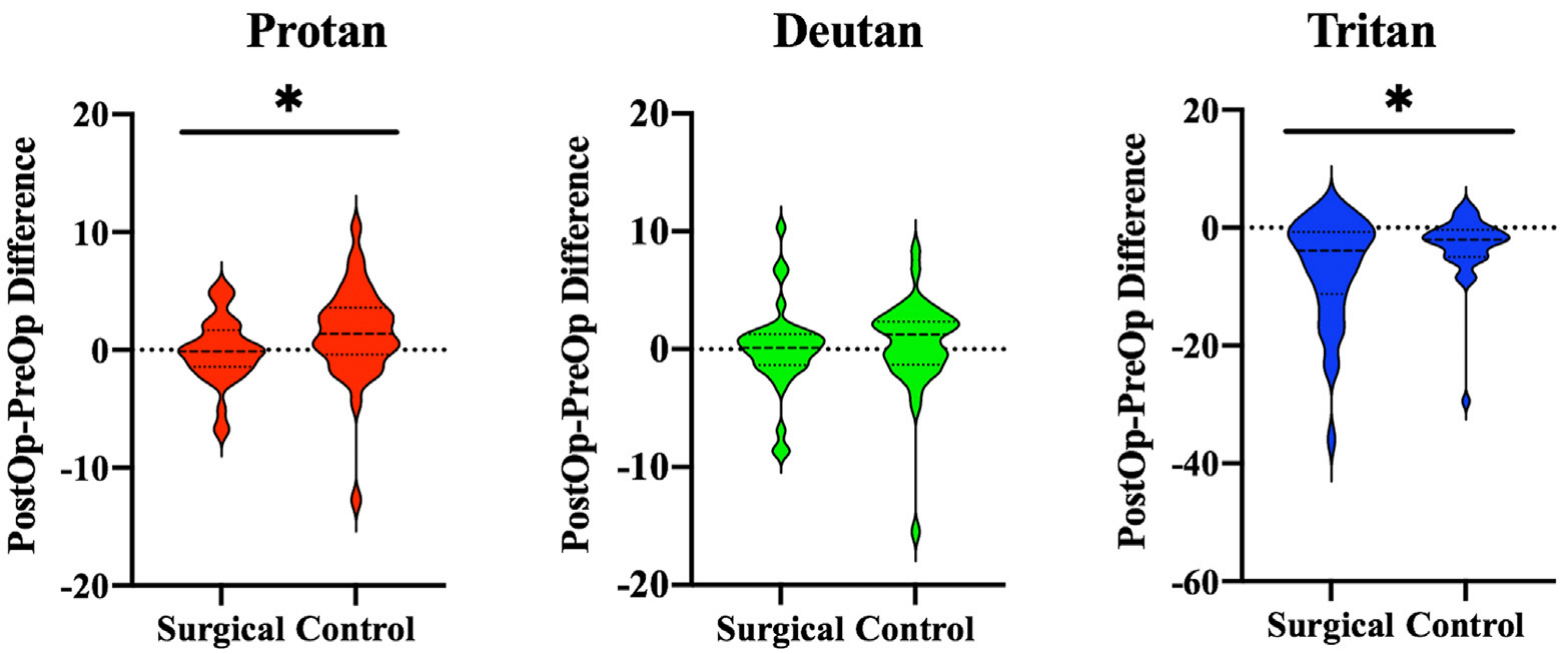
Violin plots showing the effect of cataract on Cambridge Colour Test (CCT) measurements. For each of the Trivector subtest axes, the plots compare the difference between postoperative (PostOp) and preoperative (PreOp) measurements in the surgical and control eye. The dotted lines represent the upper and lower quartiles, whereas the dashed line represents the median. Color vision measures are in × 1000 CIE1976 luv units. ^∗^Statistically significant differences between the eyes.

A Wilcoxon signed-rank test was undertaken to quantify the effect of cataract on color vision measurements. The test was completed using the difference test values before and after surgery (specifically after surgery minus before surgery) in control and surgical eyes for each of the Trivector subtest axes. Results of this test showed a statistically significant difference caused by cataract in both protan (*P* = 0.02) and tritan (*P* = 0.02) axes but not along the deutan axis (*P* = 0.13). These results represent the fact that significant change in color vision sensitivity in L and S cones is caused by cataract surgery (Fig 2).

### Can Effect of Grade and Severity of Cataract on Color Sensitivity Be Quantified

The Trivector models were run with color sensitivity measurements with independent variables set as cataract grade and cataract severity for color vision sensitivity differences for the Trivector axis. Where mixed cataract was present, that eye was given multiple grades. For all 3 models, severity of cataract was shown to have a positive slope; therefore, as severity increased, color vision sensitivity was affected to a greater extent; however, results of cataract grade (grades 1–4) all showed a negative gradient, meaning that nuclear sclerotic cataract produced the most profound effect on color vision sensitivity. However, in all 3 groups, cataract grade and color sensitivity measurements did not predict changes in color vision measurements in a statistically significant manner. For protan, the severity measurements were coefficient 0.58 (95% confidence interval [CI], –0.54 to 0.81; *P* = 0.69), and the grade measurements were coefficient –0.14 (95% CI, –0.15 to 0.51; *P* = 0.32). For deutan, the severity measurements were coefficient 0.47 (95% CI, –0.51 to 0.07; *P* = 0.74), and the grades of cataract measurements were coefficient –0.23 (95% CI, –0.17 to 0.02; *P* = 0.11). For tritan, the severity measurements were coefficient 0.25 (95% CI, –0.12 to 0.00; *P* = 0.07), and the grades of cataract measurements were coefficient 0.18 (95% CI, –0.16 to 0.03; *P* = 0.19).

## Discussion

We showed that learning to conduct the low-vision CCT Trivector test occurs between the first 2 tests, so the first test should be regarded as a learning test and discarded. We elucidated that cataract causes significant changes in the protan (*P* = 0.02) and tritan (*P* = 0.02) color vision vectors but not in the deutan axis (*P* = 0.13). All operated eyes were emmetropized as a result of cataract surgery, thereby negating chromatic aberration as a confounding variable in color vision assessment. The impact of cataract on color vision with maximum impact in the blue region was reported previously.^20^^,^^21^ Evidence for a protein-type defect previously was less robust than seen in this work. Studies in which color vision sensitivity was measured in healthy participants with a yellow filter placed over the eye did not show a significant reduction in color vision sensitivity in the same manner as seen herein with cataract.^22^ This underlines the idea that pathologic mechanisms are at work other than prereceptor absorption of shorter-wavelength light by the yellowed lens. One such possibility is oxidative stress, and this may explain the formation of cataract as a result of intraocular surgery.^23^, ^24^, ^25^ The lack of a change in the deutan axis may be secondary to the impact of the hyperopic shift in myopic eyes bringing the middle wavelengths into focus as the cataract develops. Additionally, our data showed a greater variability in the deutan axis color measurements, making it more difficult to determine a statistically significant result.

Although we were unable to elucidate a precise quantitative relationship between cataract type or grade and color vision defect, the regression models for all 3 axes did display a positive coefficient for severity, indicating that increased severity of cataract was associated with greater decrease in color vision measurements. Furthermore, the data also highlighted that nuclear sclerosis exerted the greatest effect on color vision sensitivity. The lack of a statistically significant relationship may have been indicative of the small sample size with limited statistical power in view of the prevalence of mixed cataract. It is rare for patients to seek treatment for cataract with a single cataract type, so it may not be possible to differentiate these effects in a patient population.^26^^,^^27^ Additionally, the Lens Opacities Classification System III has remained a mainstay in both clinical practice and research since the early 1990s; however, this test does not take into account specific lens features of cataract, such as water clefts, vacuoles, focal dots, and retro dots, which may affect color vision measurements in different ways.^28^^,^^29^ The change in visual acuity will not be a factor in explaining the results because the low-vision CCT is designed specifically for use in low vision and performance is independent of visual acuity. Future work would benefit from using lens densitometry to provide a more objective method for the classification of cataract. This study aimed to emulate clinically used measures.

Color vision has been used as an outcome measure in interventional trials such as gene therapy.^2^ Gene therapy involves delivery of the therapeutic vector via intravitreal or subretinal injections. This is a risk factor for the formation of cataract.^30^ Therefore, when assessing the results of clinical trials, it is useful to understand the direction of changes expected as a result of cataract to differentiate it from the therapeutic effect.

## Conclusions

From this study, we were able to elucidate that cataract has a statistically significant effect on color vision sensitivity as measured by the low-vision CCT Trivector test. A quantifiable significant effect was seen in both the protan and tritan axes but not in the deutan axis. We also established that it is not possible to disentangle the effects of a specific type and severity of cataract on color vision measurements because disease progression increases the probability of > 1 type of cataract being present within the lens. The quantifiable effect of cataract now may be taken into account in ongoing gene therapy trials using color vision as an outcome measure. This study provides information about how to account for the impact of iatrogenic cataract formation resulting from surgical techniques on color vision outcome measures.
